# School based working memory training: Preliminary finding of improvement in children’s mathematical performance

**DOI:** 10.2478/v10053-008-0083-3

**Published:** 2011-07-20

**Authors:** Marcus Witt

**Keywords:** working memory, training, central executive, mathematics, children

## Abstract

Working memory is a complex cognitive system responsible for the concurrent storage and processing of information. Ggiven that a complex cognitive task like mental arithmetic clearly places demands on working memory (e.g., in remembering partial results, monitoring progress through a multi-step calculation), there is surprisingly little research exploring the possibility of increasing young children’s working memory capacity through systematic school-based training. Tthis study reports the preliminary results of a working memory training programme, targeting executive processes such as inhibiting unwanted information, monitoring processes, and the concurrent storage and processing of information. Tthe findings suggest that children who received working memory training made significantly greater gains in the trained working memory task, and in a non-trained visual-spatial working memory task, than a matched control group. Moreover, the training group made significant improvements in their mathematical functioning as measured by the number of errors made in an addition task compared to the control group. Tthese findings, although preliminary, suggest that school-based measures to train working memory could have benefits in terms of improved performance in mathematics.

## Working memory training in primary school

### What is working memory?

 There is a lot more to working memory than the simple short-term storage of information. *Working memory* refers to a complex cognitive system that is responsible for the storage and processing of information in the short term. Although there are several models of working memory, the most widely known and the one that has proved most robust in the face of research evidence is that first proposed by Baddeley and Hitch (1974). This model consists of four parts. Two “slave” systems, the phonological loop and the visuo-spatial sketchpad, are thought to be responsible for the short-term storage of phonological and visuo-spatial information, respectively. The episodic buffer (Baddeley, 2000) is thought to integrate information in various forms into an integrated whole for a short period. These elements are connected and co-ordinated by the “central executive,” responsible for controlling and directing attention (Engle, 2002). The central executive component is thought to monitor cognitive processes, inhibit unwanted information from current processing, and to control the complex processes involved in the concurrent storage and processing of information (Oberauer, Süß, Wilhelm, & Wittman, 2003). 

### Working memory and children’s mathematics

 There is a weight of evidence suggesting that working memory is a good predictor of mathematical skills (Kroesbergen, Van de Rijt, & Van Luit, 2007; Passolunghi, Vercelloni, & Schadee, 2007; Swanson & Kim, 2007). There is also direct evidence that working memory capacity has an impact on children’s ability to perform mathematical tasks at school. Gathercole and co-workers (Gathercole & Pickering, 2000; Gathercole, Pickering, Knight, & Stegmann, 2004) found significant impairments in working memory capacity in a group of children who had scored below the expected level in national mathematics tests at age 7. The low-attaining children were particularly impaired on working memory tests requiring the simultaneous processing and storage of information. Many classroom-based mathematical tasks (such as keeping track of counting, doing mental arithmetic, or understanding a mathematical word problem) require the temporary storage of information while it is processed and/or integrated with existing information in long-term memory. Alloway, Gathercole, Kirkwood, and Elliot (2009) provide compelling evidence that children with recognised deficits in working memory experience a range of difficulties in school related to learning in general (i.e., distractability, problems generating new solutions, and monitoring the quality of work) and in particular subjects including mathematics. 

### Studies of working memory in mathematically disabled children

 Further evidence of the importance of working memory in children’s mathematical processing has been provided by studies comparing the working memory functioning of mathematically disabled children with that of normally achieving children. The persistent use of counting-based strategies, indicative of poor working memory has been found to be a feature of children with mathematical disability (MD; Geary, 1993; Gersten, Jordan, & Flojo, 2005). D’Amico and Guarnera (2005) found both central executive and visual-spatial working memory deficits in a group of children with MD only while Rosselli, Matute, Pinto, and Ardila (2006) found that groups of children with MD only and those with co-morbid reading problems had significantly lower working memory scores than controls on tasks measuring both phonological loop and central executive function. McLean and Hitch (1999) reported deficits in visual-spatial and central executive working memory in a group of children with specific impairments in mathematical processing. Van der Sluis, van der Leij, and de Jong (2005) also reported deficits in visual-spatial working memory in a group of MD children. 

 This evidence suggests that children with different forms of mathematical disability (both with and without co-morbid reading problems) perform less well than controls on working memory tasks and corroborates that stated earlier, that working memory is key to the efficient mathematical processing. While the studies cited above suggest strongly that children’s mathematical processing is dependent on working memory, the precise contribution of the different components of the working memory model to mathematical processing remains unclear. Kyttälä, Aunio, and Hautamäki (2010) found that young children with mathematical difficulties did show working memory deficits, but that these deficits were not uniform across the group. Interestingly, given that the children in their sample had not yet begun formal mathematical instruction, they suggest that poor working memory might cause poor mathematical performance. 

### The role of the phonological loop in children’s arithmetic

 There has been considerable research into the role of the phonological loop in children’s mathematical processing, but the results are not conclusive. Jordan, Hanich, and Kaplan (2003) found no connection between children’s knowledge of addition facts and their phonological working memory skills. Holmes and Adams (2006) also reported, no contribution of phonological loop scores to differences in national mathematics test scores at the end of KS 2 (when the children are 11 and move from primary to secondary school), although they did speculate that the phonological loop may be involved in retrieving arithmetical facts from long-term memory. Grube and Barth (2004) also suggested that the phonological loop was involved in basic fact retrieval. Noël, Seron, and Trovarelli (2004) found that phonological loop functioning was a better predictor of First Grade children’s addition performance 4 months later than was central executive functioning and concluded (along with Hecht, Torgesen, Wagner, & Rashotte, 2001) that phonological skills play an important role in children’s growing mathematical capabilities. The picture among children is further complicated by age. McKenzie, Bull, and Gray (2003) looked at the addition performance of children aged 6-7 years and 8-9 years under conditions of articulatory suppression, which would prevent phonological loop functioning. While the suppression of the phonological loop had a big impact on the arithmetical performance of the older children, the younger children did not suffer. This suggests that addends are encoded phonologically from around the age of 7 years onwards. 

### Visual-spatial working memory and children’s mathematics

 When considering the role of visual-spatial working memory in children’s mathematical functioning, it may well be important to take account of the precise age of the child. Young children appear to rely more on their visual-spatial working memory than do older children (McKenzie et al., 2003). This would support the notion that the acquisition of certain literacy skills (at around the age of 7) is accompanied by an ability to re-code visual stimuli into a phonological form that can be rehearsed in phonological working memory. McKenzie et al. (2003) tested children of 6-7 years and 8-9 years on addition under control conditions and with interference to phonological working memory (articulatory suppression) and visual-spatial working memory (visual noise). The performance of the younger children was highly disrupted by the interference to visual-spatial working memory, and completely unaffected by interference to phonological working memory. The older children showed a more complex pattern of disruption, with some decrement of performance under both disruption conditions. 

 This pattern of differential reliance on different components of the working memory model has also been found in a recent study by Holmes and Adams (2006). They found that a mazes memory task accounted for more variance in mathematical performance for children aged 7 and 8 years than it did for children aged 9 and 10 years. In a study using children specially screened for mathematical problems without co-morbid reading and literacy problems, D’Amico and Guarnera, (2005) found that the children scored worse on tests of visual-spatial working memory and central executive working memory than control subjects who were not mathematically impaired. The mathematically impaired children did not score worse than the controls on any of the tasks tapping phonological working memory except digit span, which uses mathematical stimuli and may therefore be affected by the robustness of the participants’ representations of numbers in long-term memory. 

 This evidence suggests that young children carry out mathematical tasks using a mental representation of the numbers involved that relies on visual-spatial working memory. This is consistent with earlier work by Hughes (1986), in which young children were shown to be better able to solve simple addition and subtraction problems presented as imagined situations rather than as abstract numbers. Holmes and Adams (2006) support this idea and suggest that visual-spatial working memory might provide a mental workspace in which children are able to represent abstract problems in a concrete, and therefore more manipulable, form. 

### The role of the central executive in children’s mathematics

 Thomas, Zoelch, Seitz-Stein, and Schumann-Hengsteler (2006) used a random generation task to see its effect on the mathematical performance (addition and multiplication) of children in primary school. The authors concluded that central executive resources were needed to answer these types of mathematical questions, but that the demands made of the central executive were lessened the more automatic the processes for mental addition and multiplication became. A random generation secondary task was also used by Seitz and Schumann-Hengsteler (2000, 2002) to investigate the effect of central executive load on simple and difficult multiplication calculations and on addition and multiplication calculations. They concluded that the central executive, but not the phonological loop, was involved in the process of retrieving information from long-term memory and in keeping track of carry operations in more complex calculations. Subsequent research (e.g., Holmes & Adams, 2006) has suggested that it is the phonological loop that is primarily responsible for the retrieval of information from long-term memory. 

 Seitz and Schumann-Hengsteler’s work supports earlier studies by Passolunghi and Cornoldi (2000) in which a group of children with poor performance in mathematical problem solving were found to be impaired in working memory tasks tapping the updating function of the central executive. These findings are supported by those from a subsequent studies (Passolunghi & Pazzaglia, 2004, 2005) in which both updating and the inhibition of unwanted information from working memory were found to be impaired in children who performed poorly in mathematical problem solving tasks. Updating the contents of working memory and the inhibition of unwanted information from working memory are closely related and are both regulatory functions of the central executive (Baddeley, 1996). 

 Several studies with children who have trouble solving mathematical problems have found deficits in their ability to inhibit unwanted information from working memory. The studies typically measure inhibition errors, where a previously seen piece of information is recalled in preference to the target information. Passolunghi, Cornoldi, and De Liberto (1999) found that poor mathematical problem solvers made more inhibition errors than controls during a working memory task. These findings were supported by further studies (Passolunghi & Siegel, 2001, 2004) in which working memory inhibition errors were made significantly more often by children identified as poor mathematical problem solvers than by controls matched for age, gender, and vocabulary. 

 The results from the studies reviewed above all point to the importance of the central executive and especially inhibition in mathematical problem solving. This is not unexpected, as mathematical problems require the assimilation of a lot of information, some of which may be irrelevant, and may therefore make much greater demands on the executive system than other mathematical tasks. Van der Sluis, de Jong, and van der Leij (2004) found evidence of deficits in inhibition and in task switching in children with global mathematical difficulties (i.e., not just in mathematical problem solving) compared to normally developing controls. McLean and Hitch (1999) also found deficits in central executive functioning among children identified as having specific impairments in arithmetic abilities rather than problem solving. These children performed more poorly on a novel “trails” task in which they had to switch retrieval strategy and to monitor their progress in terms of which strategy they had used. The authors concluded that the arithmetic-impaired children had central executive deficits related to these two specific functions. 

 The change towards the greater use of direct retrieval appears to be related to working memory capacity, with children with a larger working memory span using direct recall more often (Steel & Funnell, 2001), or at least beginning to use it earlier (Mabbott & Bisanz, 2003). This latter study found generally high correlations between the use of direct retrieval and (central executive) working memory (as measured by backwards digit recall and operation span) for children in Fourth Grade (9-10 years of age). Barrouillet and Lépine (2005) found that children with higher working memory spans were more likely to use a direct recall strategy for simple addition questions. The difference in strategy use became more pronounced as the size of the smallest addend increased. Given the apparent importance of the central executive component of the working memory model in children’s mathematical functioning, the extent to which central executive performance can be enhanced through training is of great interest to teachers. 

Given the range and complexity of the evidence about the contribution of working memory to children’s mathematical processing, but the weight of evidence supporting the involvement of the central executive, the decision was made to focus on this component of the working memory model for the training intervention.

### Working memory training

 Many of the studies into the cognitive effects of working memory training have concentrated on the populations with specific impairments in working memory functioning such as schizophrenic patients (Bell, Bryson, & Wexler 2003) and people with attention deficit hyperactivity disorder (e.g., Klingberg et al., 2005), where training has resulted in improved working memory functioning. Although it is not possible to make definitive predictions about the effects of working memory training on healthy participants on the basis of work with specific groups of patients, the existing evidence suggests that interventions do not have to be limited to the most cognitively impaired participants in order to be effective. There is some evidence that the working memory functioning of normal adults can be changed by practice (Tomasi, Ernst, Caparelli, & Chang, 2004). Several studies with healthy adults (e.g. Olesen, Westerberg, & Klingberg, 2004) have shown improvements in reaction time and/or accuracy in practiced working memory tasks. Oberauer and Kliegl (2004) found that adult participants were able to improve dual-task performance over the course of 8 to 12 weeks of training. 

 There have been few studies investigating possible gains in school-based achievement as a result of working memory gains brought about by training. There is evidence that young children who are given attention and memory training can use these skills to improve literacy levels (Posner & Rothbart, 2005). Further evidence of gains in literacy following phonological working memory training has been provided by Maridaki-Kassotaki (2002). She gave phonological working memory training to Greek kindergarten children using practice in non-word repetition. The children receiving the non-word repetition training had significantly better reading scores at the end of their first year in school than a control group. 

The research reviewed above suggests that working memory training might be a means of improving the achievement of primary school children in a number of school areas, although there has been very little research looking at this possibility in mathematics. This study sought to explore the extent to which in-school working memory training could improve working memory performance and whether any such improvement led to performance gains on a mathematical task thought to make demands of working memory.

## METHOD

### Participants

In order to address these questions a group of children aged 9 to 10 years was given a 6-week course of working memory training that focused on the central executive. The sample consisted of 38 children, from four state primary schools in the south west of England. All the children were in Year 5 (mean age 116.13 months, *SD* = 3.43 months, range 112 to 123 months) at the time of testing. There were 15 males and 23 females.

All the participating children were given measures of central executive working memory (backwards digit recall), visual-spatial working memory (visual patterns), and mathematical (addition) performance. After the initial measures, the sample was divided into a control (non-intervention) group and an intervention group who received the 6-week working memory training programme. Following the working memory training, all the participating children were re-tested and the performance of the two groups compared.

The children were drawn from four different schools in the same region of the UK. The division of the sample was done on a “matched-pairs” basis. Each child in the intervention group was matched with a child in the control group. Care was taken in matching the pairs, so that both children in each pair were from the same class. This was done to eliminate any differences in mathematical instruction within each matched pair. It also ensured that each of the four teachers from whose classes the participants were drawn had children in both the intervention and the non-intervention (control) groups. This was done to ensure that the different teachers could not influence mathematical outcomes by concentrating their teaching on specific areas of the curriculum or changing the amount of the school day devoted to mathematical instruction. The children were then matched as closely as possible for mathematical performance firstly and then for working memory performance. As gender did not prove to be an important factor in the pre-training measures, this was not considered when matching the pairs.

### Central executive task (backward digit recall)

This task was taken from the Working Memory Test Battery for Children (WMTB-C; Pickering & Gathercole, 2001). Children heard a list of digits, which they were required to repeat back in reverse order. The trials were administered in blocks of six trials with the sequence of digits increasing in length by one digit for each block. If a child scored four correct trials in any block, he/she moved to the next block. If a child failed on any three trials in a block, the task was terminated. Post-training, the same task was used, but with different strings of digits to eliminate any effects of the children learning the digit strings. In order to achieve a more fine-grained discrimination between the children’s performance, the number of correct trials is reported.

### Visual-spatial working memory (visual patterns)

Visual-spatial working memory performance was measured using an adaptation of the visual patterns task (Della Sala, Gray, Baddeley, Allamano, & Wilson, 1999). The children were presented with matrices of squares for 2 s. In each matrix, some of the squares were empty and some filled in black. The matrix then disappeared for 2 s, after which, they were shown a blank version of the matrix and asked to indicate where the filled squares had been. Each child was presented with 10 patterns that gradually increased in difficulty by containing more filled squares. For each matrix, the child had to recall the position of all the filled squares for a correct response. The total of correct responses was recorded. The same task was used for the post-intervention measure, but the patterns of filled and empty squares were changed.

### Mathematics task

The children were presented with 20 addition questions which they had to calculate mentally. The addends were visible until the child answered. All the questions required the children to store partial results while they completed the calculation. The questions were of four types:

1. The addition of three single digit numbers (this required the children to remember the result of adding two numbers while the third number was then added).

2. The addition of two double-digit numbers where there was no carrying (i.e., the sum of the units digits was not greater than 9 and the sum of the “tens” digits was not greater than 9).

3. The addition of two double-digit numbers in which there was one “carry” from the units to the tens.

4. The addition of two double-digit numbers in which there were two carries (from the units and from the tens).

The post-intervention measure was similar, but with different questions for reasons described above. The number of errors and completion time for the whole task were recorded.

### Working memory training

The working memory training detailed below was carried out over a period of 6 weeks. The children in the intervention group were seen individually by the author in a quiet location in school but outside the child’s classroom. The working memory training sessions all took place in the afternoon to minimize disruption to the children’s schooling. Each training session lasted approximately 15 min, but there was some flexibility depending on each child’s level of enthusiasm and the conversations that took place between the experimenter and the child.

#### Week 1

The children played an imagination game designed to help with remembering a string of objects. They were given a list of objects to remember (e.g., cow, boat, hat, arrow, ice-cream) and then encouraged to imagine a story in which there was a cow in a boat wearing a hat, which was hit by an arrow which then landed in an ice-cream. They were encouraged to connect mental images of the objects listed and to use imaginative or unusual ways to connect them. Having done this, they were given practice with two further lists of objects and encouraged to use mental images to link the objects together in order. Finally the children were given the chance to practise the Backward Digit Recall task. They were not explicitly encouraged to use mental imagery to help with the task, although some children reported trying to visualise the numbers that needed to be manipulated in the task.

#### Week 2

The children were introduced to the idea that repeating things “in your head” could make them easier to remember. The children were encouraged to practise remembering lists of objects forwards using a sub-vocal rehearsal technique, that is, to repeat the names of the objects internally in order to fix them in memory. The children were all able to recall more forwards than backwards and the discussion moved towards how it might be possible to use the sub-vocal rehearsal technique to make the backwards digit recall task easier. Some of the children suggested repeating the list several times forwards so that it became more fixed in their memory. A couple of children suggested repeating the list forward silently until they reached the final digit and then saying it out loud. Following this, the children were given the chance to practise the backward digit recall task again. No explicit advice was given about an effective strategy.

#### Week 3

The children practised the Backward Digit Recall task using their favoured strategy. The rest of the session focused on another central executive working memory task: the updating task. Lists of familiar objects (not digits) were read to the children who, of had to recall the three smallest. For example, from the list: “plate, car, pea, house, butterfly, thimble, guitar”, the correct response would be “plate, pea, thimble” (in any order). The children were encouraged to use either a visual chaining method, or a sub-vocal rehearsal method to make the task easier. The updating task is designed to tap the children’s ability to keep information active in working memory while they process it by comparing the sizes of the objects stored in memory with the newest object.

#### Week 4

The focus of the week was on inhibiting unwanted information from working memory (also thought to be a function carried out by the central executive component of Baddeley and Hitch’s, 1974, model). The children were presented with a series of pictures of familiar objects (banana, coin, umbrella, hammer, car, horse, ladder, cat, strawberry, etc.) using a PowerPoint presentation on a PC and asked to recall as many of the items in the list as possible in any order. After a short break, the children were presented with a similar task. In addition to the to-be-remembered stimuli, each slide contained a number of distracting items (attractive pictures of biscuits, chips, sunsets, fireworks, etc.).

All the children found the second version of the task more difficult than the first and all were able to recall the distracting items. This allowed some discussion about the kinds of things that could stop them from using memory accurately. The children were then presented with a second attempt at the “with-distractors” version of the task and asked to concentrate hard on not letting the distracting pictures prevent them from remembering the other pictures. Strategies for preventing distraction were discussed.

#### Week 5

The children practised a counting recall task. This task requires participants to store information in the face of a concurrent processing demand. The children were presented with collections of shapes to be counted on a PC screen. The children were told to remember the results of the counts and then to repeat them back in order after the final count. The level of working memory demand was manipulated by the number of counts that had to be remembered. Each child practised the task for between 5 and 10 min depending on their level of enthusiasm. The number of counts to be recalled was set at a level that the children found challenging, but manageable. The level of difficulty was increased as the children became more proficient at the task and wanted to challenge themselves. There were several trials prepared at different levels or working memory demand, so that the task could be adapted to the working memory ability of individual children.

#### Week 6

The final week of the intervention returned to practise the backward digit recall task that the children would be tested on the following week. There was a chance for the children to ask questions and to discuss with the author what they felt they had gained from the intervention and the strategies that they had found most useful. The post-training testing took place one week after the working memory intervention had finished.

## RESULTS

There were no significant correlations between the children’s age and any of the mathematical or working memory measures (all *rs* < .30, *ps* > .05). Independent samples *t*-tests showed that there were no significant sex differences in performance on any of the working memory tasks or the mathematical tasks. One-way ANOVA revealed no significant differences in the working memory or mathematical performance of the children from the different schools (for all measures *F* < 1.5, p > .20).

The comparison of the intervention and non-intervention (control) groups was done using matched-pairs *t*-tests. These were thought to be statistically more robust than independent samples *t*-tests of the two groups. The matched pairs *t*-tests showed that there were no significant differences in any of the pre-intervention working memory or mathematical scores between the intervention group and the non-intervention (control) group. The two groups were matched very closely for addition errors. The intervention group scored slightly better than the non-intervention group on the visual patterns task, and slightly worse on the backward digit recall task. None of these differences approached statistical significance. Post-training comparisons of the two groups (intervention and non-intervention) revealed that there were significant differences in performance on the visual patterns task, the backward digit recall task and addition accuracy. There were also statistically significant differences between the groups in terms of the changes in performance between the pre- and pos*t*-tests. An improvement in performance between the pre- and post-intervention measures is shown as positive and a decrement in performance is shown as negative. The results show clearly that the group that received the intervention made statistically greater gains in both the backward digit recall task and in their mathematical accuracy than the control group (see Table 1).

**Table 1.** Scores for the Intervention and Control Groups Before and After the Intervention and the Change in Scores.

Post-intervention measures	Time	Intervention group	Control group	*t*	*p*	Effect size *d*
Visual patterns (correct trials)	Before	7,95	7,74			
	After	8,68	7,37	2,59	*p* < .05	0,93
	Change	0,73	-0,37	1,84	*p* < .05	0,65
Backward Digit Recall (correct trials)	Before	11,11	12,16			
	After	17,05	13,00	2,31	*p* < .05	0,65
	Change	5,94	0,84	3,99	*p* < .001	1,25
Addition time (in seconds)	Before	149	163			
	After	148	168	-0,98	*ns*	0,33
	Change	1	-5	0,94	*ns*	0,35
Addition accuracy (number of errors)	Before	3,26	3,32			
	After	1,58	2,95	-2,63	*p* < .05	0,76
	Change	1,68	0,37	3,37	*p* < .01	0,69

*Note*. The table shows scores before and after the intervention as well as the change in scores. An improvement is shown as a positive change score and a fall in performance is shown as a negative change score. Effect sizes are reported for the “after” and “change” comparisons, that is, for those showing the effects of the intervention.

## DISCUSSION

The group that received the intervention had significantly better post-intervention visual-patterns scores than their matched controls. One explanation for this result is that the working memory intervention has had a significant “knock-on” effect into other areas of working memory. There is also considerable support (e.g., Rudkin, Pearson, & Logie, 2007) for assuming a close connection between visual-spatial working memory and the central executive. The results from this study do not contradict that contention, as practice on a central executive task appears to have had a significant impact on non-practised visual-spatial task performance. There is also some previous evidence (e.g., Klingberg et al., 2005) that working memory intervention can have benefits on non-trained tasks.

An alternative, but related explanation is that some of the children who received the working memory training were using visual strategies to recall the digits. This strategy lends itself well to the backward digit recall task. Two of the children talked of having ”discovered” the strategy of imagining the digits and placing them in their imagination from right to left as they were read out. The children then “read back” what they could see in their mind’s eye from left to right. The children who favoured a visual strategy could have been using their visual working memory during the course of the intervention and might therefore have been expected to show improved performance on the visual patterns task.

 Even more encouraging was the finding that the group that received the central executive working memory training had better mathematical performance than the non-intervention group after the intervention. These differences were in terms of lower error rates rather than increased speed, suggesting that the working memory training did more than simply increase the children’s processing speed. It may have enabled the children to monitor their own performance more effectively. This is a highly important finding, as it suggests that programmes aimed at training children’s working memory could potentially lead to gains in mathematical performance. Kyttälä et al. (2010) found working memory deficits in young children who had not yet begun formal instruction in mathematics, leading them to suggest that poor working memory causes poor mathematical performance. 

It could be that the use of digits as stimuli in the practised working memory task (backward digit recall) somehow helped the children to improve their performance on the addition task. There is nothing inherently quantitative in the way the digits are used in the task. The digits used are 1 to 9 and, given the nature of the addition questions, the numbers that would need to be stored during concurrent processing would always be more than 9. However, it may be that frequent practice of a task using digits might make the encoding of digits more robust and therefore somehow help with the addition task. This in itself would be an interesting finding. A further study is planned in which the practised central executive task does not use digits.

Could there be other explanations for this finding? It might be the case that the children who had had the intervention felt more comfortable with the experimenter, or more comfortable carrying out working memory tasks as a result of the time spent with the experimenter during the previous weeks. This possible reduction in anxiety might have been responsible for the improved scores. The children may have been under the impression that they were expected to perform better, which may have led them to try harder on the task than the children in the control group. Both these explanations can be questioned. Many of the children who took part were known to the experimenter and would have been very comfortable with him. All the children showed very high levels of motivation. It could be argued that the children in the intervention group might have been growing tired of practising working memory tasks and would have had reduced levels of motivation at the time of the post-training testing.

In trying to explain the improved mathematical performance, a good understanding of the ways in which central executive working memory works to facilitate addition performance is important. All of the 20 addition questions in the task involved either carrying or the retention of one part of the calculation while the other part was computed. The improved post-intervention error rates for the children in the intervention group suggest that their working memory system was better able to deal with these demands accurately (Thomas et al., 2006). These children were better able to maintain the partially calculated parts of the calculation while they were doing other calculations. This would have been the case for both double-digit calculations and for all those requiring carrying. This result supports the suggestion that the central executive is involved in relatively simple addition (Jordan et al., 2003).

What these findings are not able to show is the precise way in which practice in the backward digit recall task has led to improved performance. It was clear from watching the children carry out the task that some of them found different and more efficient strategies to complete the task. However, there was no overall pattern among the children and the range of strategies that the children adopted was wide. Some of the children repeated the number string forwards a number of times to fix it in their memory, were then silent for a few seconds before repeating the whole string in reverse order. Other children repeated the string forwards silently to themselves until they reached the last item that they had not so far said. They then said this item out loud. For the children who adopted this strategy it was successful. There were several children in the intervention group who were very resistant to this strategy even when encouraged to use it.

These findings suggest that changes in strategy use do have an impact on the working memory performance of children. However, many of the children in the intervention group (all but one of whom improved their performance on the task) did not appear to implement a new or different strategy. This suggests that, for some children at least, improvement is possible by a better or more accurate execution of an existing strategy. It could be that there is some component of working memory that is “trainable” beyond improvements in strategy use.

The findings from this study are clearly provisional and more research is needed to explore further the possibility that direct training can boost children’s working memory functioning. Further research is also needed to examine the possibilities for improvement in mathematical functioning brought about as a result of working memory training. Specifically, this study gives no information about the durability of any gains made. It has not explored the possibility of using working memory training with groups of children in a classroom. There is also the possibility of Hawthorne Effects in this study (i.e., the fact that the children in the intervention group made performance gains purely as a result of being the focus of some additional adult attention). While such effects could have accounted or the improvements in working memory scores, it seems less likely that the secondary effects of improved mathematical accuracy would have been the result of experimenter interest. However, this possibility can not be ruled out and further research is needed.

The lack of evidence about durability should not however detract from the current findings. There are many phenomena where continued intervention or practice is necessary in order to produce and secure long-term gains in performance. The fact that this is the case does not render such interventions futile or impossible to carry out. There are many possibilities for incorporating working memory training into daily classroom activities, both in mathematics lessons and in other areas of the curriculum. Many primary schools in the UK now have some time each day devoted to the promotion of “thinking skills”.

The results of this study provide provisional and qualified evidence that school-based working memory training can, in the short-term at least, boost children’s working memory performance possibly by a more accurate use of mnemonic strategy, or by boosting the child’s ability to retain and manipulate information accurately. Importantly, the study suggests that this might have an impact on school tasks such as mental addition, the accurate performance of which places demands on central executive working memory. Clearly more research is needed to explore the durability of these improvements in performance and the possibility of carrying out working memory training with groups or even whole classes of children. It is not possible, based on this study, to separate any benefits that came from improved strategy use and those that might have come from a genuine increase in working memory capacity. Further research is needed to explore this question in order that working memory training that is done in schools in the future can be made as effective and efficient as possible. 
